# Dynamic Transmit Profile Selection in Dense Wireless Networks

**DOI:** 10.3390/s21010134

**Published:** 2020-12-28

**Authors:** Łukasz Kułacz, Adrian Kliks, Paweł Kryszkiewicz, Bartosz Bossy

**Affiliations:** Institute of Radiocommunications, Poznan University of Technology, 61-131 Poznan, Poland; adrian.kliks@put.poznan.pl (A.K.); pawel.kryszkiewicz@put.poznan.pl (P.K.); bartosz.bossy@put.poznan.pl (B.B.)

**Keywords:** adaptive transmission, brain-inspired communications, dense wireless network, energy consumption

## Abstract

The development of wireless networks can be characterized by both the increased number of deployed network nodes as well as their greater heterogeneity. As a consequence, the distance between the neighboring nodes decreases significantly, the density of such a wireless network is very high, and it brings to the mind the analogy to the human brain and nervous system, where a highly simplified scheme of information delivery is applied. Motivated by this similarity, in this paper, we study the possibility of the application of various transmission profiles in order to optimize the overall energy consumption in such dense wireless networks. The transmission profile specifies the radio access and energy consumption of the wireless transceiver (network node), and is characterized by the tuple of parameters, e.g., the total transmit power or minimal required signal-to-noise ratio (SNR). In the considered multi-hop network, we assume that each node can be set to the most promising transmission profile to achieve some predefined goals, such as (sensor) network reliability or transmission energy efficiency. We have proposed the new graph-based routing algorithm in such a dense wireless network, where total power consumption of message delivery is minimized by multihop and multimode transmission. The theoretical definition of the prospective transmission schemes is supported by the analysis of the results of the simulation experiments.

## 1. Introduction

One of the noticeable trends in the development of contemporary wireless networks is the continuous densification of wireless networks by an increasing number of network nodes, such as access points, base stations, radio remote heads, or simply transmission points [1,2,3]. Moreover, some of the nodes can act only as relays. This means that the significant reduction of mean inter site distance (ISD) is observed, which in turn may result in improvement of the observed signal to noise ration (SNR) at the receiver side or decreased energy required for transmission. Moreover, when the number of nodes is extremely high (like in ultra dense networks, UDN), the immediate consequence is that typically the direct line-of-sight (LOS) communication link can be created between most of the neighboring nodes. This prevents fading effects from appearing. This observation is particularly applicable in the context of wireless sensor networks (WSN), where the number of active transmitting and receiving nodes may be very high. In such a network, it is of high importance to route all messages with low latency, high reliability, but also with high energy efficiency, as the problem of overall energy consumption is crucial. Various approaches can be found in the literature [4,5,6,7]. Mainly, in [4], the authors have considered the ultra-dense networks operating in the millimeter frequency band, where the base stations are assumed to be able to harvest energy. Moreover, various additional constraints have been analyzed, such as presence of interference and requirements on the expected quality of service. The authors have proposed the iterative gradient algorithm for joint user association and power optimization problem. Next, in [5], the authors have discussed the problem of interference and energy management in ultra-dense networks, however focusing on heterogeneous schemes. Again, the iterative and distributed solution has been considered. Similarly, in [6], the interference aware algorithm has been proposed for energy efficiency maximization in ultra-dense networks. The authors have observed that the cooperation opportunities between the nodes increase with the density of deployed nodes. In this context, the cooperative game has been proposed, called cooperative energy efficiency maximization game. The performance of this algorithm has been verified concerning energy efficiency, but also fairness expressed by the Jain’s index.

One may notice that the structure of such a future wireless network brings to mind the human nervous system (HNS) and human brain, where millions of nervous cells are connected in order to deliver messages along the human body [8,9]. Although the number of neurons is huge, at the same time, the processing of information in a single link is highly simplified, as no complicated coding is applied, i.e., channel coding for improving data reliability. For example, one of the existing information coding schemes in HNS is to modify the frequency of sent pulses as a function of the importance of information. This highly simplified scheme of information delivery between two points of HNS leads to the conclusion that in dense wireless networks, the application of low-complicated processing algorithms could be a viable solution increasing networks’ energy efficiency and transmission reliability. In [10], it is discussed how the transmission functions observed in HNS can be mapped to wireless communications technologies.

Following the above-mentioned inspiration, we will consider a dense wireless network. Because of varying transmission condition of each link, e.g., resulting from varying path loss, the optimal (e.g., from energy efficiency or reliability perspective) radio access technology can vary. We will consider both computationally simple transmission schemes, i.e., analog modulations, and more sophisticated digital transmissions utilizing redundancy codes. While the optimization of transmission profile, i.e., modulation and demodulation scheme that is characterized e.g., by the energy consumption of the wireless transceiver (network node) and signal-to-noise ratio (SNR) required for correct data reception, has been reported in [11] for a single link, here, the network-level perspective will be investigated. We propose a scheme for selection of nodes for information transmission and their profile selection such that the energy-efficiency of the dense wireless network can be optimized while keeping reliability at a high level. In particular, we consider the case when the deployed wireless nodes may operate (i.e., transmit and/or receive) in two modes, i.e., an analogue-like and a digital one, in order to deliver a message to the succeeding node. In the conducted computer experiments, we perform the analysis of the efficiency of the proposed algorithm for transmission profile selection. The novelty of the paper can be summarized as follows:

- we have identified four operating modes of the wireless nodes in dense wireless network, called hereafter transmission profiles, extending the initial proposal presented in [12]; these modes can be classified as combinations of analogue and digital way of signal processing at the receiver and transmitter part, i.e., mainly analogue-analogue, analogue-digital, digital-analogue, and digital-digital,
- An algorithm has been proposed that allows one to minimize the power consumption of a message passing by utilization of multihop and multinode transmission. The algorithm selects the proper nodes and their configurations (transmit power and mode). It is an extension of prior work [11], where such optimization was performed at link level. In this paper, we have investigated the route optimization scheme;
- we have proposed an heuristic to chose the links to be included in the multihop route using their scaled length. The result of this heuristic is close to the optimal solution obtained using brute force algorithm.
- we have shown that the multimode and multihop transmission provide advantage over the single hop transmission in terms of the consumed power. However, there is an optimal numer of hops over which the power consumption increases.

The remainder of the paper is organize as follows. In Section 2, the recap of the brain inspirations toward the dense wireless communications is provided, followed by the presentation of the assumed system model and identification of the research problem. In Section 3, the power consumption and signal quality models for the considered transmission profiles are described. The proposed algorithms for the relay nodes and their transmission profiles selection are discussed in Section 4, whereas the simulation scenario and achieved results are presented in Section 5. The paper is concluded at the end.

## 2. Energy Efficiency in Brain-Inspired Wireless Dense Networks

### 2.1. Brain Inspirations for Wireless Communications

Within the HNS, the main component is the neuron. The message is travelling within the neuron from the cell body along the axon to the dendrites and then it is conveyed between the neighboring neurons by means of neurotransmitters [8,10]. The new neuron is activated only when the stimulus is strong enough to fire the neuron and cause new, so-called, action potential. When the action potential reaches axon’s end then synapses can release new neurotransmitters. Those neurotransmitters can be received by further neurons. Different neurotransmitters can stimulate or inhibit subsequent neurons. Basically, we can highlight three types of neurons: motor neurons, sensor neurons and inter neurons. Motor neurons are located, e.g., in muscles and enables muscle contraction. Sensor neurons are located, e.g., in skin and enables one to sense touch. Inter neurons is a type of neuron which connects other types of neurons. Neurons are responsible also for our thoughts, dreams and intelligence.

Information in nervous system transmitted by neurons is coded by so-called rate coding. It means that messages can have a different level of importance for organism. In this case, more important information is transmitted with higher rate of impulses, e.g., information about pain. On the other hand, less important information is transmitted with a lower rate of impulses, e.g., information of smell. Additionally, more important signal can suppress less important signal.

Projecting such a transmission scheme to the dense wireless communication networks, one may think of a highly simplified transmission scheme where messages are transmitted without any advanced channel coding for data protection, where it is assumed that data blocks are delivered correctly when the requirement for minimum SNR value is fulfilled. In other words, the link between two nodes will be treated as reliable when the distance between them is small enough to guarantee reception of the signal above the minimum required SNR.

### 2.2. Considered System Model and Problem Identification

In our research, we consider the brain-inspired dense wireless network consisting of *N* nodes deployed randomly over certain area. The density of this network is intentionally so high that there exists a line-of-sight (LOS) between any two neighboring nodes. At the same time, the distance between these nodes is assumed to be small enough that very low transmit power can be applied to deliver information to the neighbors. Each of the nodes may act as a source of the message (origin), the destination of the message (the node, where the message has to be delivered), or as relay (which only forwards the received message). In consequence, the whole network of wireless nodes may be treated as the transmission (forwarding) plane for reliable message delivery between any two nodes within this network. We assume that each wireless node possesses the ability to receive data and if necessary—to transmit it further. It is assumed that the message to be transmitted is of analog nature, e.g., measured voltage or recorded voice. As such, the transmission can be digital (requiring analog-digital and digital-analog conversion) or analog. This allows one to compare both analog and digital transmissions in the same framework. Each wireless relay node may operate in one of four transmission modes:

- fully analogue (AA), when both reception and transmission consider analogue modulations;
- fully digital (DD), when both reception part and transmission part consider digital modulations;
- analogue-digital (AD), when the reception side implements analogue signal demodulation, whereas the transmit part uses digital modulations;
- digital-analogue (DA), when the reception side implements digital signal demodulation, whereas the transmit part uses analogue modulation.

In addition to the four possible configurations of relaying nodes, also the source and the destination nodes may operate in analog or digital modes. Moreover, in addition to the four above-mentioned transmission modes, we can consider each rely in two sub-modes in terms of data processing inside it. Mainly, the well known amplify-and-forward (denoted hereafter as AF) and decode-and-forward (marked as DF) schemes are applied. In the former case, the input signal is amplified as well as the all the incoming distortions and noise. In the DF case, the signal is first decoded (so to some extent the distortions are removed here), and then encoded again. Each mode may be characterized by the set of parameters and functions describing the behaviour of the node if working in a certain mode. The fixed parameters are, for example, the maximum allowed transmit power $P_{T,mode}^{\max}$, minimum possible transmit power $P_{T,mode}^{\min}$, total power consumption $P_{mode}$, or minimum required signal-to-noise-ration ${\mathrm{SINR}}_{mode}^{\min}$ etc., where mode∈{AA,AD,DA,DD}. In terms of functions describing the instantaneous behaviour of the node in the given mode, one can identify the function describing total consumed power or the function defining the minimum square error in terms of signal-to-noise ration. These functions are derived later in the paper. In order to deliver the message efficiently in terms of energy consumption between the source and destination, the route shall be selected that minimizes the total power consumption while keeping transmission reliability at assumed level. As show in [11,12], the selection of the best transmission scheme (analogue or digital) depends on the distance between the neighboring nodes. Thus, in this paper, we address the problem of energy-efficient path selection, where each relay node may choose to work in one of the four above-mentioned transmission modes. Please note that the selection of the best routing path is particularly challenging, as the cost of each hop in the network is changing with the total number of utilized hops. This is due to the fact that we assume that the message will be correctly received at the destination node only when the overall signal quality at the receiver will be above some threshold. However, each wireless node that participates in data forwarding from the source to destination provide deterioration of the signal quality. For example, let us imagine that the overall acceptable loss in modulated signal MSE equals *X*. In case of direct data transmission between source and destination (direct link, one hop), the whole loss may be caused by this link. However, when *n* hops are assumed, the whole error budget has to be divided between links. In average in each hop only X/n of the total error budget can be observed. Thus, the higher the number of hops the higher quality of each link is required. In the following sections, we propose the heuristic solution to this problem.

## 3. Power Consumption and Signal Quality Models

In this section, the transmission modes available in our system are presented. In general, analog and digital transmission between nodes is allowed but transmission modes can be changed according to channel conditions and power consumption in each hop. Therefore, the power consumed by each allowed combination of relaying has to be described [11]:

- analog-analog (AA) relay—in this case, the analog modulated signal (we consider frequency modulation—FM) is received, amplified and then fed to the antenna. The analog relay does not bring the signal to baseband because any signal reconstruction is not possible from the FM modulation point of view, i.e., it will not improve the signal quality. Thus, the total power consumption model of the AA relay is given by:
  (1) $$\begin{array}{c} P_{\mathrm{AA}}=P_{\mathrm{PA}}+P_{\mathrm{CA}}^{\mathrm{TX}}+P_{\mathrm{CA}}^{\mathrm{RX}}=\frac{4}{\pi }\sqrt{P_{\mathrm{TX}}\cdot PAR\cdot P_{T}\left(t\right)}+P_{\mathrm{CA}}^{\mathrm{TX}}+P_{\mathrm{CA}}^{\mathrm{RX}}, \end{array}$$
  where $P_{\mathrm{PA}}$ is the power consumption of the power amplifier and $P_{\mathrm{CA}}^{\mathrm{TX}}$ and $P_{\mathrm{CA}}^{\mathrm{RX}}$ are the constant powers consumed by circuit components of the transmitter and the receiver, respectively. For class B power amplifier, $P_{\mathrm{PA}}$ depends on the average transmission power $P_{\mathrm{TX}}$, the peak to average ratio PAR and transmission power at time t, i.e., $P_{T}\left(t\right)$. Knowing the distribution of the instantaneous waveform power, it can be easily calculated [13]. In the case of FM modulation, we are dealing with a fixed envelope, thus PAR=1 and $P_{\mathrm{PA}}=\frac{4}{\pi }P_{\mathrm{TX}}$.
- analog-digital (AD) relay—in this mode, the received FM signal is demodulated. Then, the signal is fed to the transmitter using digital modulation, specifically QAM. Thus, the power consumption of the AD relay consists of the power consumption of the FM receiver and the QAM transmitter:
  (2) $$\begin{array}{c} P_{\mathrm{AD}}=\underset{\mathrm{FM}\;\mathrm{receiver}}{\underset{︸}{P_{\mathrm{FMdemod}}+P_{\mathrm{CA}}^{\mathrm{RX}}}}+\underset{\mathrm{QAM}\;\mathrm{transmitter}}{\underset{︸}{P_{\mathrm{ADC}}+2P_{\mathrm{DAC}}+P_{\mathrm{enc}}+P_{\mathrm{PA}}+P_{\mathrm{CD}}^{\mathrm{TX}}}}. \end{array}$$
  The power consumption of the FM demodulator is described by $P_{\mathrm{FMdemod}}$. The QAM transmitter consumes power $P_{\mathrm{ADC}}$ while converting analog modulating signal to digital domain, $P_{\mathrm{DCA}}$ in each of the utilized digital-analog converters in the quadrature modulator, $P_{\mathrm{enc}}$ for the forward error correction (FEC) encoder. The constant power consumed by circuit components of the QAM transmitter is given by $P_{\mathrm{CD}}^{\mathrm{TX}}$. In the case of the digital transmission, the same power consumption model of $P_{\mathrm{PA}}$ is applied. Nevertheless, it can be observed that the values of PAR and instantaneous power distribution can be different in both modes, thus the power consumption can be different, as well. Based on datasheets of the ADC and DAC produced by analog devices, the power consumption model has been proposed. From analysis in [11], it turns out that the consumed power mainly depends on the number of channels, the device architecture and the sample rate $R_{\mathrm{samp}}$ of the ADC and DAC. Based on real values of power, consumption of the ADC and DAC is given by:
  (3) $$\begin{array}{c} P_{\mathrm{ADC}}=7.719\cdot 10^{-6}\cdot {R_{\mathrm{samp}}}^{0.6036}, \end{array}$$
  (4) $$\begin{array}{c} P_{\mathrm{DAC}}=8.219\cdot 10^{-5}\cdot {R_{\mathrm{samp}}}^{0.447}. \end{array}$$
  The power consumption of the FEC encoder is usually modeled as $P_{\mathrm{enc}}=\xi_{\mathrm{enc}}R$, where *R* is the achieved link-throughput, while $\xi_{\mathrm{enc}}$ is the computational efficiency of the FEC encoder in $W/\left(\mathrm{bit}/s\right)$. This relatively simple model not only has been applied in many papers focusing on the energy efficiency optimization [14,15,16], but it has also been confirmed by measurements in [17].
- digital-analog (DA) relay receives the signal in the form of a QAM modulated waveform which, after processing, is fed to FM transmitter. Thus, the total power consumption model of this mode consists of the QAM receiver and the FM transmitter:
  (5) $$\begin{array}{c} P_{\mathrm{DA}}=\underset{\mathrm{QAM}\;\mathrm{receiver}}{\underset{︸}{2P_{\mathrm{ADC}}+P_{\mathrm{DAC}}+P_{\mathrm{dec}}+P_{\mathrm{CD}}^{\mathrm{RX}}}}+\underset{\mathrm{FM}\;\mathrm{transmitter}}{\underset{︸}{P_{\mathrm{FMmod}}+P_{\mathrm{CA}}^{\mathrm{TX}}}}, \end{array}$$
  where $P_{\mathrm{CD}}^{\mathrm{RX}}$ is the constant power consumed by QAM receiver components, $P_{\mathrm{FMmod}}$ describes the power consumption of the FM modulator and $P_{\mathrm{dec}}$ denotes the power consumption of the FEC decoder which is (similar to encoder) modelled as $P_{\mathrm{dec}}=\xi_{\mathrm{dec}}R$. The computational efficiency of the FEC decoder in $W/\left(\mathrm{bit}/s\right)$ is defined by $\xi_{\mathrm{dec}}$. While two AD modules are needed by the quadrature demodulator architecture, the DAC is needed to generate the demodulated analog signal.
- digital-digital (DD) relay—in this case, the received as well as the transmitted signal uses digital QAM modulation. Nevertheless, the received signal can be only amplified and forwarded to destination or can be demodulated and decoded to the form of bits and then back encoded and modulated. Thus, for the digital relay, two submodes can be distinguished:
  ○ amplify and forward (AF) relay, of which the power consumption is described by equation:
    (6) $$\begin{array}{c} P_{\mathrm{AF}}=P_{\mathrm{PA}}+P_{\mathrm{CD}}^{\mathrm{TX}}+P_{\mathrm{CD}}^{\mathrm{RX}}=\frac{4}{\pi }\sqrt{P_{\mathrm{TX}}\cdot PAR\cdot P_{T}\left(t\right)}+P_{\mathrm{CD}}^{\mathrm{TX}}+P_{\mathrm{CD}}^{\mathrm{RX}}, \end{array}$$
  ○ decode and forward (DF) relay with the total consumption power given by:
    (7) $$\begin{array}{c} P_{\mathrm{DF}}=\underset{\mathrm{QAM}\;\mathrm{receiver}}{\underset{︸}{2P_{\mathrm{ADC}}+P_{\mathrm{dec}}+P_{\mathrm{CD}}^{\mathrm{RX}}}}+\underset{\mathrm{QAM}\;\mathrm{transmitter}}{\underset{︸}{2P_{\mathrm{DAC}}+P_{\mathrm{enc}}+P_{\mathrm{PA}}+P_{\mathrm{CD}}^{\mathrm{TX}}}}. \end{array}$$
    It can be observed that in the DF mode, the analog signal reconstruction (using DAC) and digitization (using ADC) can be omitted. This step will consume additional power with no gain in the signal quality.

In the case of the source and the sink of the signal, the transmitter and receiver can work in the analog or digital mode. Thus, the power consumption by the digital transmitter $P_{\mathrm{TX}-D}$ and by the digital receiver $P_{\mathrm{RX}-D}$ is equivalent to the power consumed by QAM transmitter:

(8) $$P_{\mathrm{TX}-D}=P_{\mathrm{ADC}}+2P_{\mathrm{DAC}}+P_{\mathrm{enc}}+P_{\mathrm{PA}}+P_{\mathrm{CD}}^{\mathrm{TX}},$$

and QAM receiver:

(9) $$P_{\mathrm{RX}-D}=2P_{\mathrm{ADC}}+P_{\mathrm{DAC}}+P_{\mathrm{dec}}+P_{\mathrm{CD}}^{\mathrm{RX}},$$

respectively. An analogous situation occurs when the transmitter and/or receiver works in the analog mode. In this case, the power consumed by analog transmitter $P_{\mathrm{TX}-A}$ is equal to power consumption of the FM transmitter:

(10) $$P_{\mathrm{TX}-A}=P_{\mathrm{FMmod}}+P_{\mathrm{CA}}^{\mathrm{TX}},$$

and the power consumed by the analog receiver $P_{\mathrm{RX}-A}$ is equivalent to the FM receiver:

(11) $$P_{\mathrm{RX}-A}=P_{\mathrm{FMmod}}+P_{\mathrm{CA}}^{\mathrm{TX}}.$$

Remarkably, in our system, the transmission mode is selected according to channel conditions for each link. Therefore, in the case of multi-hop transmission, it is possible that transmitter and receiver work in different modes.

The above formulas require first to calculate the required TX power. However, the TX power has to be adapted to the channel conditions and the required signal quality at the link sink. As mentioned in the previous section, multi-hop transmission requires signal quality in each component link to be slightly higher in order to obtain the required signal quality at the final receiver. In order to compare the digital and analog systems, the quality of signal is measured by an MSE value of the demodulated signal, e.g., after digital demodulation, DAC processing is required to reconstruct signal that is compared with initial modulating signal using MSE metric. Therefore, for both systems, the MSE as a function of the signal to noise ratio has been estimated using curve fitting toolbox provided by MATLAB and simulation results obtained in [11]. In the case of digital transmission, the MSE for the 16-QAM modulation with turbo coding (code rate $R_{\mathrm{cod}}=1/3$) and the bandwidth expansion equal to 12.4077 is estimated by the below equation:

(12) $$\begin{array}{c} MSE_{\mathrm{QAM}}=0.15\cdot \mathrm{erfc}\left(\frac{SNR\left[\mathrm{dB}\right]-4.98}{0.48}\right)\;\mathrm{for}\;SNR\left[\mathrm{dB}\right]\in \langle 0;\;6.5\rangle , \end{array}$$

while, for the analog transmission by equation:

(13) $$MSE_{\mathrm{FM}}=\left\{\begin{array}{ccc} 0.05\cdot \mathrm{erfc}\left(\frac{SNR\left[\mathrm{dB}\right]-1.98}{4.24}\right) & \mathrm{for} & SNR\left[\mathrm{dB}\right]\in \langle 0;\;11.25)\\ 14.21\cdot e^{-1.07\cdot SNR\left[\mathrm{dB}\right]}+2\cdot 10^{-4}\cdot e^{-0.21\cdot SNR\left[\mathrm{dB}\right]} & \mathrm{for} & SNR\left[\mathrm{dB}\right]\in \langle 11.25;\;20\rangle \end{array}\right.,$$

where $\mathrm{erfc}\left(\cdot \right)$ is the complementary error function. Knowing the required final MSE and the number of hops, MSE required at the end of each link is calculated. The ultimate goal would be to use for the edge cost, the power consumption of each link. Unfortunately, this value is unavailable at this stage of optimization. However, it is well known that typically the longer the transmission distance the higher the power consumption. Therefore, somehow weighted distance should be used as a cost of a given edge in the considered network graph. By numerical evaluation, the required SNR can be calculated for each mode. This is further mapped, knowing the wireless channel gain, to the required TX power.

## 4. Proposed Solutions

In order to allow for successful source-sink message passing the optimization algorithm has to find: set and order of interconnecting nodes (relays), the power transmitted from each transmitting node and the type of the transmission used (digital or analog). We consider that all nodes utilize the same signal bandwidth. As such, multi-hop transmission delays signal reception at the final receiver proportionally to the number of hops in comparison to direct transmission. However, as it is assumed that in this kind of a network the delay can be tolerated, the optimization goal is to minimize power consumption while providing sufficient reception quality at the end receiver (measured by an MSE value).

Such a network could be potentially optimized by representing each transmitter/receiver/relay as a single node in a graph with edge being characterized by power required to transmit (whole consumed power) through a given link (value is dependent on the pathloss between both node). The minimum power route could be found using Dijkstra algorithm. The multi-mode operation of a given link could be reflected by changing a single physical node and a single physical link into many virtual nodes (e.g., reflecting analog and digital transmissions of different configuration) and many virtual edges (connecting various nodes in various modes).

Unfortunately, such a model cannot be used as the required MSE at the end of each hop depends on the total number of hops and the order of a given hop in transmission chain. These data are outputs of a Dijkstra algorithm, unknown before optimization. Therefore, another optimization strategy has been proposed. It is composed of two phases: (1) nodes selection, and (2) transmission mode selection.

The first stage results in a selection of transmission paths. In the perfect case, it can be a single path, possibly an optimal one, or a set of paths to be considered in the second phase. Maximum, i.e., exhaustive search, is a set of all possible paths between the considered message source and sink starting from a single hop to maximum number of hops in a given network. The only possibility that can be omitted are loops, passing many times through the same node. While an exhaustive search guarantees consideration of the optimal path, it is prohibitively computationally complex for a network consisting of more than a few nodes. Therefore, some heuristics have to be proposed to find one or a few paths that can be tested in phase two for minimum consumed power. The proposed heuristic is to mark each edge between two nodes by a chosen natural power of distance (in space) between these nodes and run the Dijkstra algorithm to find path minimizing sum of such scaled distances. Natural choice of the distance exponent is 1, as it will minimize the total route distance between source and sink. However, as the received power reduces with the 2nd, 3rd or 4th power of distance in typical pathloss models, these exponents should be tested as well. The higher the used exponent, the lower probability of choosing long haul link. Utilization of the infinitive power of the distance will be equivalent to finding the path of minimum distance of the longest link.

**Figure 1:**
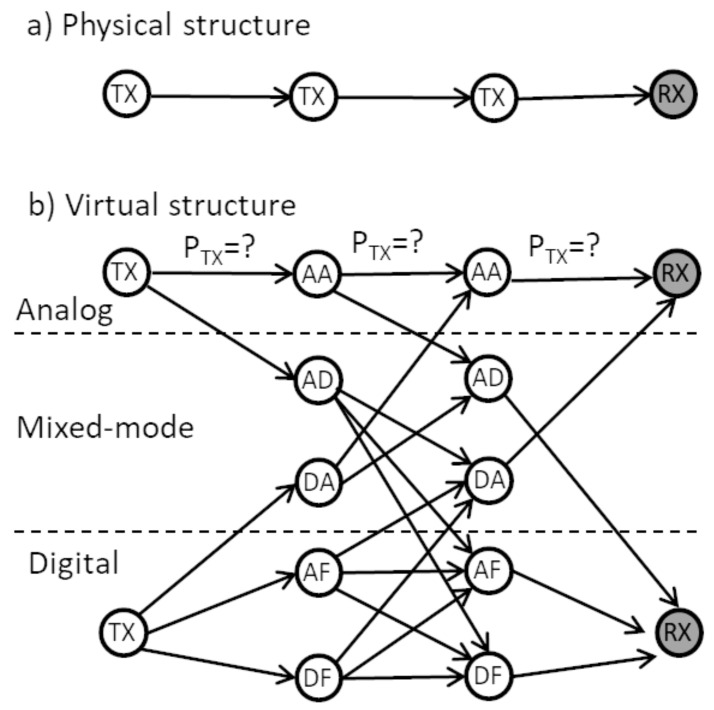
Diagram of the physical and virtual structure of the optimized subnetwork. AA: analog relay, AD: analog-digital relay, DA: digital-analog relay, AF: amplify and forward digital relay, DF: decode and forward digital relay.

The second stage is executed separately for each route defined in the first phase. Now, the physical path has a line topology and the required optimization is limited to finding correct mode on each link and transmission power. The order of nodes, distance between them and the required MSE is fixed as a result of the previous phase. As the required MSE at each receiver is fixed, the above-mentioned algorithm utilizing Dijkstra algorithm and virtual nodes and edges can be executed. An example of such a route virtualization is shown in Figure 1. The physical path is composed of one transmitter, two relays and one receiver (3 hops). The virtual structure is built using 14 nodes. While at the top analog transmitter, receiver and relays are depicted, at the bottom, fully digital nodes are depicted. In the middle part, mixed-mode relays are depicted utilizing analog-digital or digital-analog mode conversion. Each edge in this graph can be characterized by the power consumed by transmission and reception. The Dijkstra algorithm can be run to find minimum power consuming route, i.e., mode of each transmitter, receiver and relay. Observe that the analog signal source can be merged virtually with digital signal source keeping the same number of outgoing edges, i.e., 5. The same can be done with receiving edges. This results in a single start and end node simplifying optimization.

For each route specified in phase one, a single power consumption figure is obtained (in phase two). The algorithm is finalized by choosing the route utilizing the smallest amount of power.

## 5. Simulation Setup and Results

**Figure 2:**
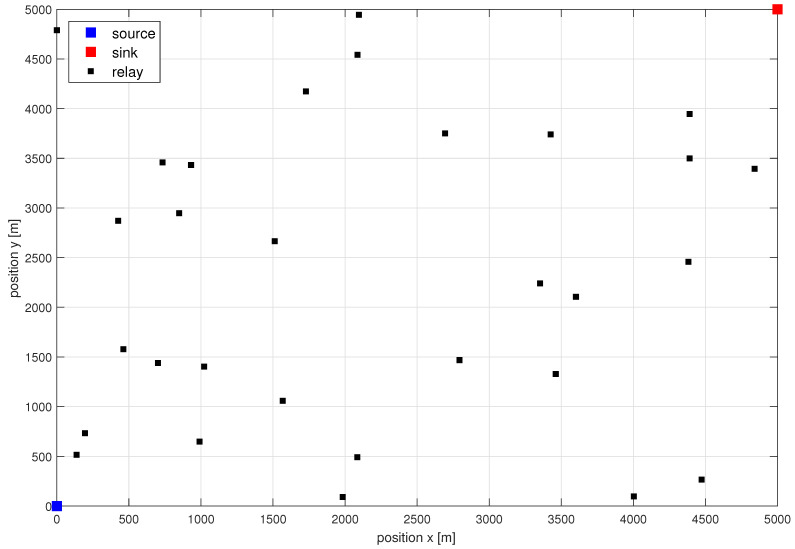
Example of the network topology with 30 relays, for area width of 5 km.

The authors considered a dense ad hoc wireless network located on a square surface with the side L=3 km, 5 km or 7 km. For each of the 1000 random network topologies analyzed, 30 network relays located according to uniform process. Traffic source is placed in one corner of the analyzed area, and traffic sink is placed in the opposite corner. Radio parameters assumed in the simulation were as follows [11]: center frequency of system $f_{c}$ was equal 3.5 GHz, and bandwidth of analyzed voice signal $B_{sig}=15$ kHz. The bandwidth expansion is equal to 12.4077. For the digital transmitter, $\xi_{\mathrm{enc}}$ equals 0.1 mW/Mbps. For the receiver, $\xi_{\mathrm{enc}}$ equals 0.8 mW/Mbps. Authors assumed $MSE=10^{-4}$ to be a fixed goal in all modes and for all number of hops at the final receiver. Additionally, the constant power used by digital transmission and FM modulation/demodulation is $P_{CD}^{TX}=P_{CD}^{RX}=135$ mW and $P_{FMdemod}=P_{FMmod}=135$ mW, respectively. The utilized pathloss model follows assumptions used in [18]. Similarly, noise power in the receiver is calculated as thermal noise increased by noise figure of 5 dB [18]. Example of the network topology is shown in Figure 2.

**Figure 3:**
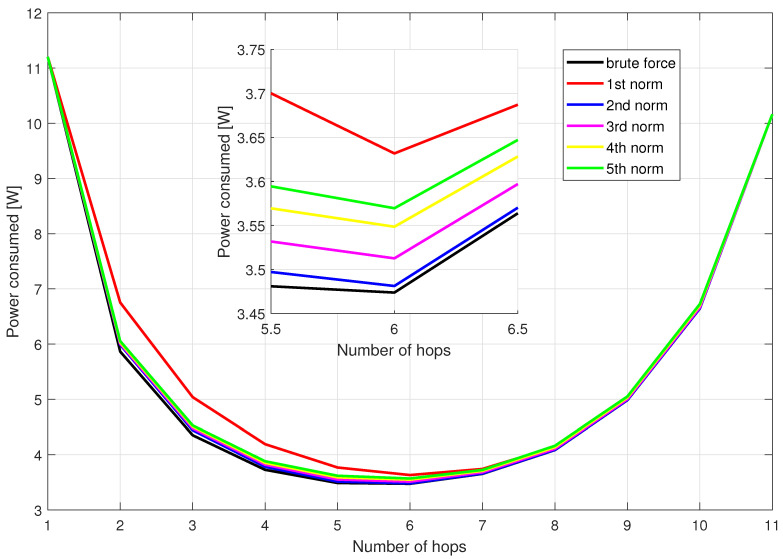
Power consumption of the network with 10 relays for area width of 5 km.

The authors conducted brute force simulations to find the best solution in terms of minimizing power consumption. Due to the high computational complexity of such a simulation, the number of relays in the network had to be limited to 10 (smaller than the standard 30 nodes). Simulations have been carried out for all considered area sizes (3, 5, 7 km). Each iteration of the simulation consisted in choosing random position of relays and checking all combinations of nodes creating paths of different length (number of hops). For each path with a fixed length and a list of used nodes, a transition graph was created (showing various transmission methods—i.e., analog, digital, mixed, similar to the example in Figure 1) and the path with the lowest power consumption was selected using the Djikstra algorithm. In the Djikstra algorithm, each possible path uses exactly the same nodes, however, by duplicating nodes to model an analog or digital relay, it was possible to select the appropriate transmission modes. The results of the discussed simulation for the area of size 5×5 km are shown in Figure 3. It shows power obtained for the considered number of hops after Dijkstra-based optimization of the transmission mode, averaged over 1000 considered random nodes locations. The simulation results for the rest of area sizes look very similar. The minimal power consumption was achieved in all the areas with 6 hops. It equals 1.555 W, 3.474 W and 6.132 W for the area width of 3 km, 5 km and 7 km, respectively. As expected, the higher the distance between sorce and destination of the message, the higher consumed power. More interesting is the advantage of the multihop transmission visible in Figure 3. The brute force algorithm shows that there is an optimal number of hops. For the direct connection, more than 3 times higher power is required. Moreover, a number of hops that is too high (higher than 6) causes the power consumption to rise again.

In addition to the brute force solution, the heuristic proposed to choose route in the first phase of the proposed algorithm in Section 4 with the distance exponent of 1, 2, 3, 4 and 5 was tested.

The purpose of testing these metrics was to find a faster way to select a path (the optimal one is found using brute force simulation). From the presented results, we can observe that for all path lengths, the average value of the power consumed is the lowest for the solution found with the brute force method as expected. The path chosen by the metric with distance exponent of 2 is the closest to this solution in each case (each number of hops). The heuristic using first distance exponent in this case gives the worst results. Exactly the same conclusions are made in case of area width of 3 and 7 km. It is worth emphasizing at this stage that these metrics were only used to choose the path between the message source and its destination. The stage of creating the virtual subnetwork graph and finding the optimal transmission modes using the Dijkstra algorithm remains unchanged.

**Figure 4:**
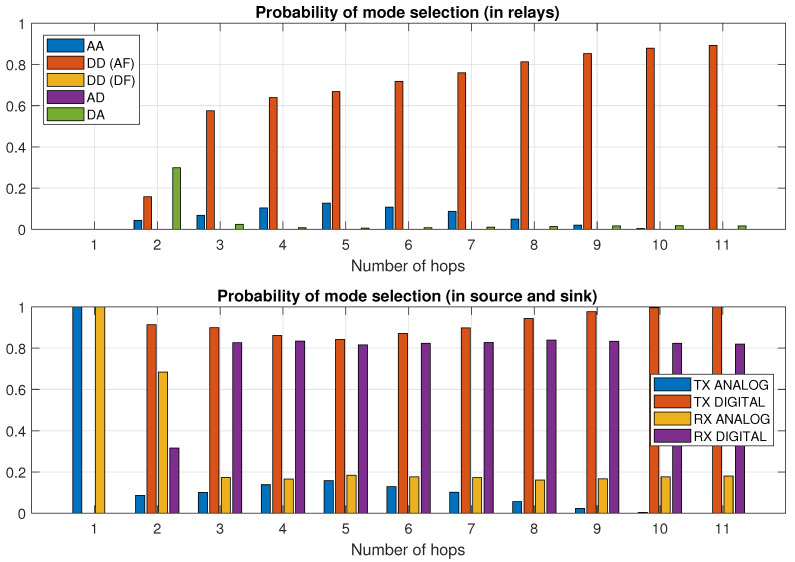
Probability of transmission mode selection (area width of 3 km).

In Figure 4, the probability of transmission mode selection is presented for network area width of 3 km. In the upper part of the figure, we can observe that in some cases (up to 10 percent of cases), analog relay is selected. However, the main part of transmission is realized through digital (amplify and forward) relay. In the lower part of this figure, we can observe the probability of transmission mode selection for transmitter (first node in the chain) and receiver (last node in the chain) separately. We can observe that for a low number of hops (e.g., 2 hops), there is a high chance for digital transmission and analog reception, but if more hops are used, the most probable receiver will also use digital transmission. For the optimal number of hops for this network, i.e., 6, both digital and analog transmission are in use.

**Figure 5:**
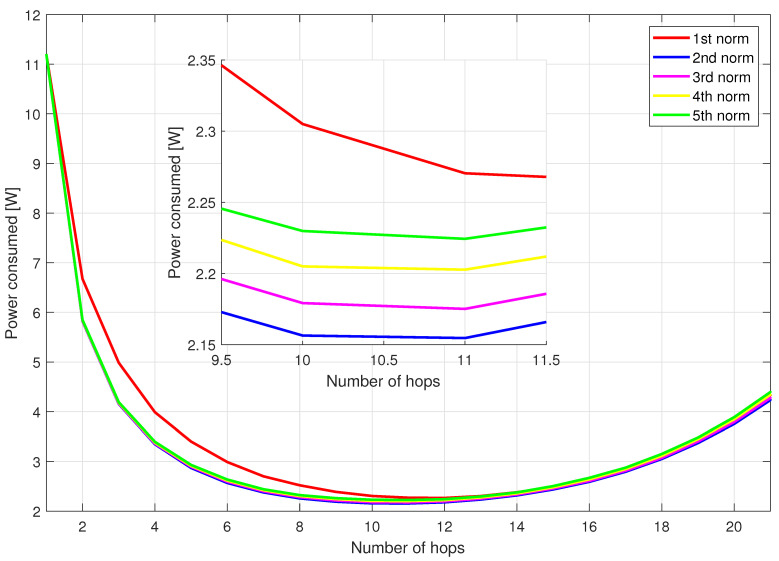
Power consumption of the network with 30 relays for area width of 5 km.

In Figure 5, the mean consumed power is showed in the network with area size 5×5 km with a full number of 30 relays distributed in this area. Due to computational complexity, there is no brute force result provided. However, paths selected using the heuristic with 5 different distance exponents behaves in the same way, i.e., the lowest power consumption is achieved using second distance exponent metric in the process of path selection.

The minimal consumed power is observed at 11 hops for the metric utilizing second power of distance. It equals 1.014 W for area width of 3 km, 2.155 W for area width of 5 km, and 3.733 W for the area width of 7 km. Again, in all these cases, there are a number of hops that minimize the power consumption and both analog and digital transmission should be available to provide highest network energy savings.

## 6. Discussion

In this paper, we have optimized the energy consumption of the dense wireless network when there is a flexibility in transmission profile selection. Mainly, following the inspirations from observing the functioning of the human-brain nervous system, two transmission modes have been compared—an analogue and digital one. The transmission modes can be changed according to channel conditions and power consumption in each hop. Therefore, each wireless relay node may operate in one of four transmission modes: fully analogue, fully digital, analogue-digital or digital-analogue. Moreover, for the fully digital mode, we distinguish the amplify and forward and decode and forward relaying. The power consumption models for each transmission mode have been proposed based on literature review, datasheets and measurements. Then, the algorithm minimizes the energy consumption based on network graph has been proposed. Achieved simulation results have shown that for direct connection the selection of the highly simplified mode (i.e., the analogous one) leads to better energy usage for achieving the same network reliability. However, the most energy efficient mode requires coexistence of digital and analog transmission within a multihop path. Let us note that the selection of the MSE value in simulations is somehow arbitrary. It is done to show some numerical confirmation of the performance of the proposed concept. Clearly, the proposed algorithms will work also for any other target value of the MSE, especially since the MSE in a function of required SNR is provided in the analytical form, in this paper.

The presented results strongly depend on the definition of the transmission profiles and the power consumption models. For example, by changing the amount of power allowed for data processing, e.g., encoding, decoding, one will affect the cost of digital transmission changing possibly the optimal solution. Nevertheless, the presented power consumption models are universal enough that adapting them to other implementations should be relatively simple. Moreover, it can be observed that the presented results confirm the hypothesis that in the case of short distances between nodes, the power consumption of the signal processing in the digital modes can dominate over transmission power and the simple transmission modes are beneficial from the energy consumption point of view.

## Data Availability

Data is contained within the article. Another data used in this study are available on request from the corresponding author.
